# Nonlinear THz Generation through Optical Rectification Enhanced by Phonon–Polaritons in Lithium Niobate Thin Films

**DOI:** 10.1021/acsphotonics.3c00924

**Published:** 2023-08-28

**Authors:** Luca Carletti, Cormac McDonnell, Unai Arregui Leon, Davide Rocco, Marco Finazzi, Andrea Toma, Tal Ellenbogen, Giuseppe Della Valle, Michele Celebrano, Costantino De Angelis

**Affiliations:** † Department of Information Engineering, University of Brescia, Via Branze 38, 25123 Brescia, Italy; ‡ National Institute of Optics—National Research Council (INO-CNR), Via Branze 45, 25123 Brescia, Italy; § Department of Physical Electronics, Fleischman Faculty of Engineering, Tel-Aviv University, 69978 Tel-Aviv, Israel; ∥ Department of Physics, Politecnico di Milano, Piazza Leonardo da Vinci 32, 20133 Milano, Italy; ⊥ Istituto Italiano di Tecnologia, 16163 Genova, Italy

**Keywords:** lithium niobate, THz generation, optical rectification, THz phonons, phonon-polaritons, localized surface phonon-polaritons

## Abstract

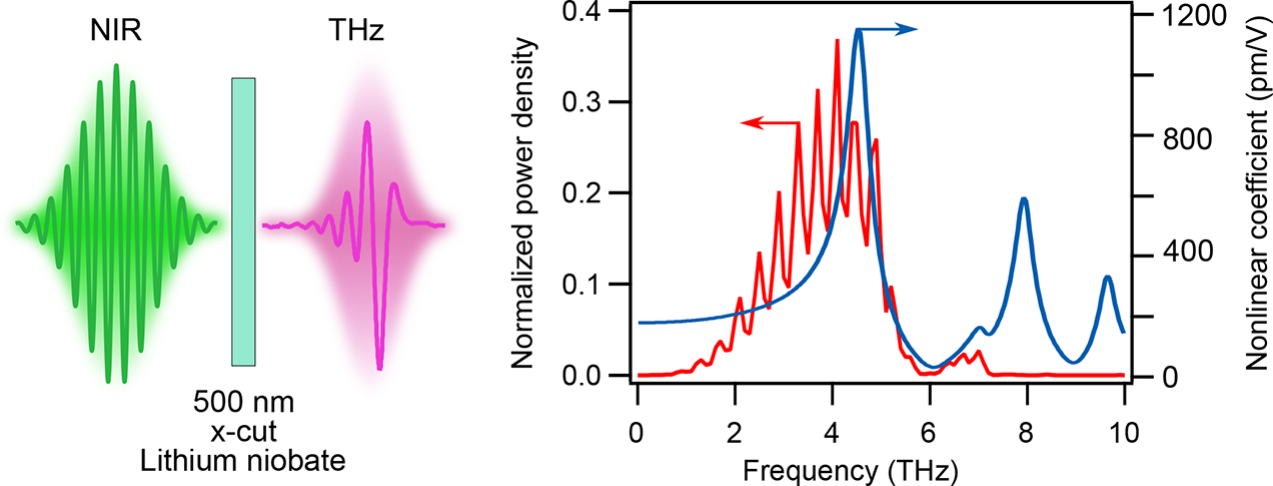

We investigate nonlinear THz generation from lithium niobate films and crystals of different thicknesses by optical rectification of near-infrared femtosecond pulses. A comparison between numerical studies and polarization-resolved measurements of the generated THz signal reveals a 2 orders of magnitude enhancement in the nonlinear response compared to optical frequencies. We show that this enhancement is due to optical phonon modes at 4.5 and 7.45 THz and is most pronounced for films thinner than 2 μm where optical-to-THz conversion is not limited by self-absorption. These results shed new light on the employment of thin film lithium niobate platforms for the development of new integrated broadband THz emitters and detectors. This may also open the door for further control (e.g., polarization, directivity, and spectral selectivity) of the process in nanophotonic structures, such as nanowires and metasurfaces, realized in the thin film platform. We illustrate this potential by numerically investigating optical-to-THz conversion driven by localized surface phonon–polariton resonances in sub-wavelength lithium niobate rods.

## Introduction

Generation and detection of electromagnetic radiation at terahertz (THz) frequencies (1–10 THz) are becoming increasingly important in various fields that have already a significant impact on society, such as medical imaging, security, and high-speed wireless communication.^1−3^ Transition of this technology from the research level to real-world scenarios requires efficient, compact, and affordable sources and detectors of THz radiation. In this perspective, solid-state THz source technologies that are being developed have the potential to fulfill several of the aforementioned requirements. Among them optical rectification of femtosecond laser pulses in nonlinear crystals has shown the ability to achieve high-intensity and broadband THz radiation,^3−6^ while nanoscale structures have the potential of providing miniaturized configurations of THz devices.^7−15^

Optical rectification is a nonlinear optical process in which an intense optical laser pulse is used to generate a low-frequency charge motion in a nonlinear medium, resulting in the emission of THz radiation. The efficiency of this process strongly depends on the properties of the nonlinear medium, the characteristics of the optical pulse, and the phase-matching condition.^2^ In this framework, lithium niobate (LN) has attracted much interest due to its high nonlinearity and high damage threshold that are associated with its broad transparency window (350–5000 nm).^16^ Nevertheless, a major issue that limits the conversion efficiency is the group velocity mismatch between optical and THz frequencies. Although in LN crystals phase-matching can occur only at some specific frequencies,^17^ tuning the phase- and group-velocity-matching condition at any desired frequency has been made possible by the tilted pulse front technique, which opened the possibility to achieve a high optical-to-THz conversion efficiency.^18,19^ However, the strong intrinsic absorption in the THz band due to optical phonon resonances has so far limited the generation of radiation above 4 THz. Interestingly, the same phonon modes also provide an enhancement of the second-order nonlinearity of the material, which is up to 2 orders of magnitude larger compared to the nonlinearity that can be attained in the optical range,^20^ and was recently exploited for efficient difference-frequency generation below 1 THz.^8^ Theoretical studies of LN-based waveguides predicted that phonon contributions may also enhance the generation of higher frequency THz radiation by optical rectification of femtosecond laser pulses.^21^

In recent years, the emergence of the integrated thin-film LN (TFLN) technology led to many new developments and applications.^22^ Hybrid gold THz antennas on TFLN circuits demonstrated promising results for integrated tailored THz sources in the lower THz frequency range (<1 THz).^23^ The TFLN platform holds great promise also for the development of efficient nonlinear metasurfaces.^16^ These may provide the ultimate flexibility in terms of spatiotemporal control of the generated THz signals, as recently demonstrated using plasmonic metasurfaces.^12,14^ In the context of THz applications, metasurfaces can be employed to control the amplitude and phase of optical pulses, which can then be rectified to produce THz radiation.^12^ However, due to their planar nature (i.e., a few hundred nanometers thickness), the interaction length is limited and mechanisms to enhance the optical-to-THz conversion efficiency are needed.^7,24^

Despite the large increase of the nonlinear response due to optical phonon modes, the strong intrinsic absorption associated with this band has so far hindered the experimental observation of efficient optical-to-THz conversion in bulk LN crystals above 4 THz. Conversely, thin LN films offer the opportunity to limit the self-absorption of the generated THz signal. In this work, we demonstrate that optical phonons can indeed significantly enhance THz generation between 2 and 8 THz by performing measurements and numerical calculations of optical rectification of an ultrashort pump pulse in LN samples with different thicknesses. The increase in the nonlinear response due to the phonon–polaritons is up to 2 orders of magnitude larger compared to the highest value that can be reached at optical frequencies. These results, along with the high damage threshold of LN compared to plasmonic materials, may drive the development of optically transparent metasurfaces for ad-hoc generation and detection of THz radiation covering the so-called “THz gap” (0.1–10 THz).^1^

## Results

**Figure 1:**
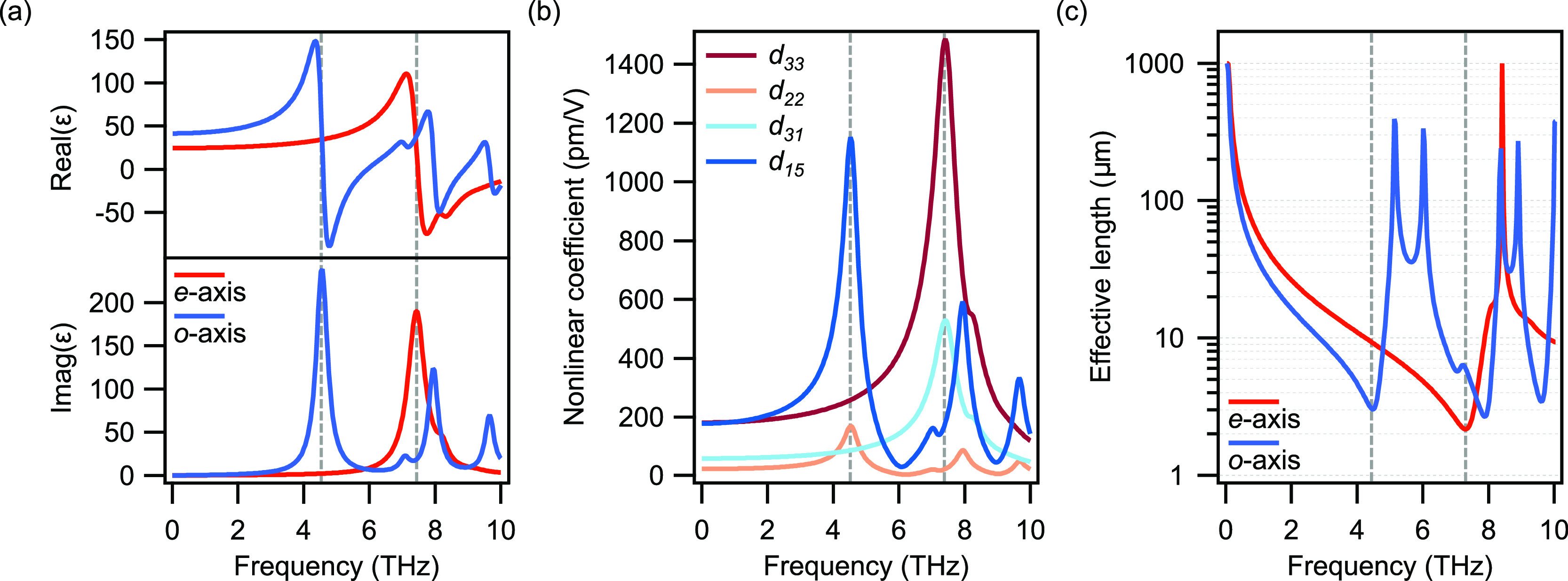
(a) Real (upper panel) and imaginary (lower panel) part of the LN permittivity for ordinary (*o*) and extraordinary (*e*) axes. (b) Nonlinear coefficients obtained from the generalized Miller’s rule model with data from ref (20). (c) Effective length of LN for *e*-axis and *oo*-axis THz generation. The vertical dashed gray lines indicate the frequency of the first phonon modes for either axes.

Polar crystals like LN possess vibrational lattice modes (i.e., optical phonons) that can couple to impinging electromagnetic radiation at THz frequencies. The dispersion characteristics of the electromagnetic wave traveling through such crystals are modified giving origin to a mixed mode known as a phonon–polariton (PhP).^25^ The complex relative permittivity function in this band is strongly affected by the PhP and it can be described by a multi-oscillator model
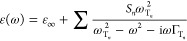
1where  is the phonon resonant frequency,  is the phonon damping rate, ε_∞_ is the high-frequency permittivity limit, and *S*_*n*_ is the oscillator strength.^25^ The value of ε(ω) obtained from eq 1 for the ordinary and extraordinary LN axes using the data reported in ref (26) (see Supporting Information S1) is shown in Figure 1a.

To understand and predict the THz radiation generated by optical rectification, we are interested in the tensor of the second-order nonlinear coefficients that, in contracted notation, assumes the form^20^
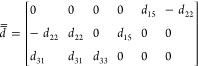
2However, only limited data for the values of these nonlinear coefficients for THz generation are available in the literature and those that can be found are relative to the low sub-THz frequency range. Due to the presence of optical phonon resonances in the spectral range of interest, we estimate the dispersion of the nonlinear coefficients by applying a generalized version of Miller’s rule that accounts for both the electronic and the ionic contributions to the nonlinear response^20,21^
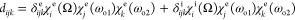
3where χ^i^ (χ^e^) is the ionic (electronic) contribution to the linear susceptibility, Ω is the THz frequency, ω_o1_ and ω_o2_ are the frequencies of the optical pulse, and δ^e^ and δ^i^ are fitting coefficients. The nonlinear coefficients of LN that are estimated from eq 3, using electro-optic experimental data at microwave frequencies^20^ to determine δ^i^ and δ^e^ are reported as a function of frequency in Figure 1b. We observe that the strongest nonlinear response is obtained when all the electric field components are aligned parallel to the extraordinary (*e*) axis, a condition that corresponds to a predominant *d*_33_ coefficient. This element of the nonlinear tensor is enhanced by a phonon resonance at 7.45 THz. Noteworthy, its value is up to 2 orders of magnitude larger than the one achieved at optical frequencies.^16,21^ A similar frequency-dependent behavior is observed for the *d*_31_ element describing a resulting THz nonlinear polarization that is oriented along the *e*-axis. Nonlinear mixing generating a nonlinear THz polarization oriented along one of the ordinary (*o*) axes, which is accounted for by the *d*_22_ and *d*_15_ elements, is instead enhanced by the *o*-axis phonon resonance at 4.5 THz.

Although this is promising for optical-to-THz conversion, the optical phonon resonances that enhance the nonlinear response also result in subsequent absorption of the THz radiation, thereby hindering this enhancement in thick LN crystals. The optimal thickness to benefit from the PhP enhancement of the nonlinear response can be estimated by calculating the effective nonlinear length, , that is defined by^27^
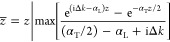
4where Δ*k* = *k*_THz_ – (*k*_o1_ – *k*_o2_) is the momentum mismatch, α_T_ is the absorption coefficient in the THz band, and α_L_ is the absorption coefficient in the optical pulse band. The result of eq 4 is shown in Figure 1c for THz generation along the *e*-axis and *o*-axis, respectively. We observe that the effective length drops drastically with increasing frequency to about 2 μm at 4.5 THz (*o*-axis) and 7.45 THz (*e*-axis), strongly limiting the broadband THz generation by thick films and obscuring the contribution of the ionic enhancement of the nonlinear interaction. Thus, to leverage the PhP enhancement of the nonlinear response, LN films with a thickness smaller than 2 μm shall be employed.

**Figure 2:**
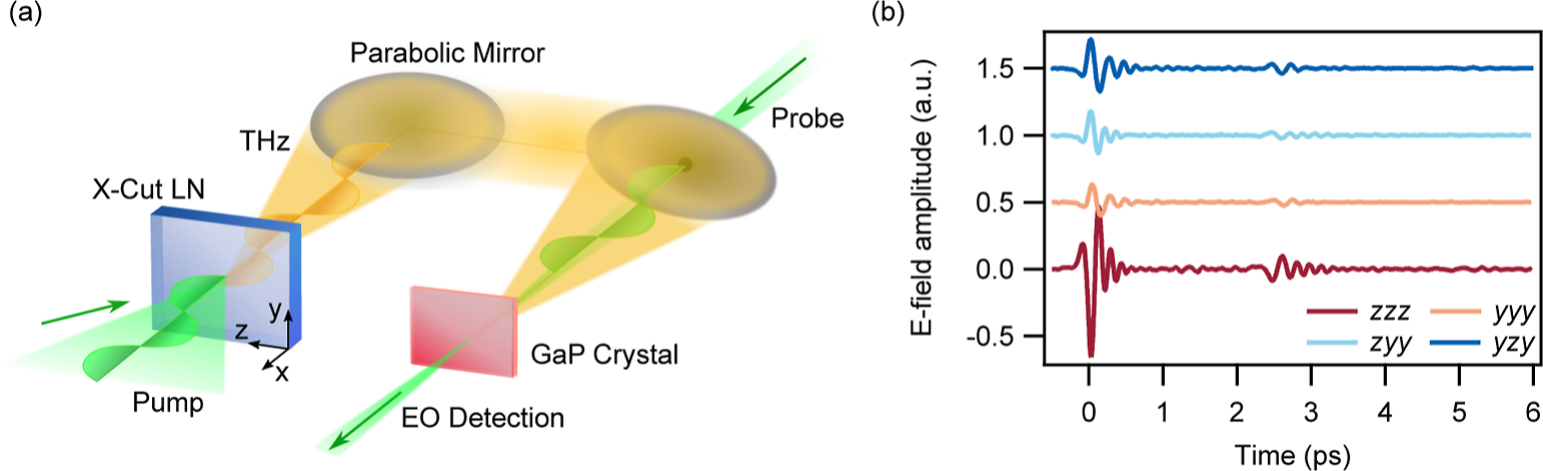
(a) Experimental setup for the generation and detection of broadband THz pulses from x-cut LN films based on time-domain spectroscopy. (b) Time domain signals from a 500 nm thick LN film which have been vertically displaced for illustrative purposes. The first letter indicates the THz signal polarization, and the second and third letters identify the pump polarization relative to the LN crystal axes.

Based on these observations, we measured the broadband THz radiation generated by optical rectification of near-infrared femtosecond pulses in thin—0.5 and 5 μm—x-cut LN films on a 2 μm thick silica layer over a 500 μm quartz substrate (NanoLN) and bulk x-cut LN crystals—83 and 133 μm (University Wafer). The experimental setup is based on time domain pump–probe spectroscopy (see Supporting Information S2) and is schematically illustrated in Figure 2a.

The LN films are pumped with linearly polarized femtosecond pulses (central wavelength of 1500 nm, pulse temporal duration of 50 fs) and the generated broadband THz pulses are temporally sampled in a GaP electro-optic crystal (100 μm thickness, response up to 8 THz, see Supporting Information S2). A synchronized probe pulse (800 nm, 35 fs) reveals the complete temporal and spectral properties of the electric field in the THz spectrum. Importantly, the polarization of the pump beam and of the probe pulse are controlled to perform polarization-resolved measurements and investigate the tensorial nature of the LN nonlinear optical response. We selected four combinations for the pump and THz polarization that allow us to isolate the contributions of the different nonlinear coefficients (see Supporting Information S3). In particular, we explore the case of an optical pulse where the electric field is aligned with the *z*-axis of the LN and the THz radiation is polarized parallel to the same axis (*zzz*); optical pulse electric field parallel to the *y*-axis of the LN and THz radiation parallel to either *y*-axis (*yyy*) or *z*-axis (*zyy*); optical pulse electric field at 45° from the *z*-axis of the LN and THz radiation parallel to *y*-axis (*yzy*).

**Figure 3:**
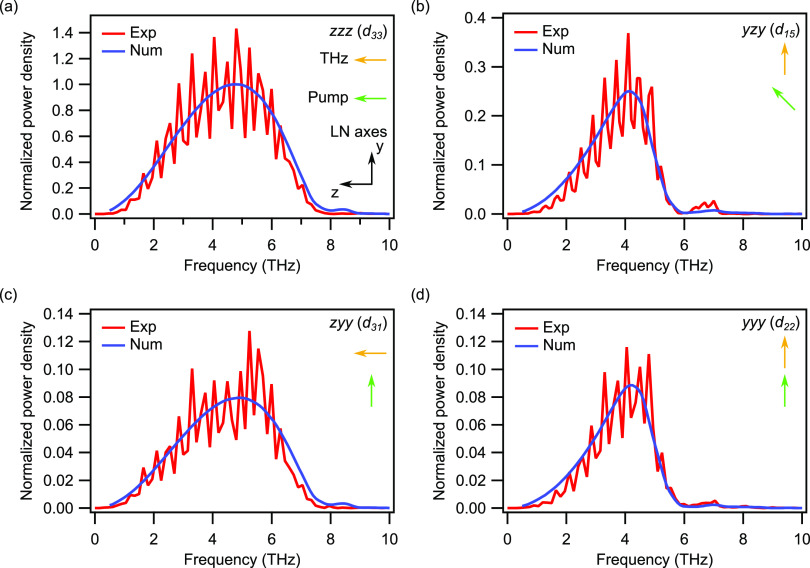
(a–d) THz emission spectra from an LN film of 500 nm thickness calculated numerically or derived from the measurements. The last two letters indicate the optical laser pulse polarization with respect to the LN axes, and the first letter indicates the polarization of the emitted THz radiation. The mild fringes in the experimental spectra are due to the echo pulses in the time trace. The numerically calculated spectra are filtered by the GaP electro-optic crystal response.

We first measured THz generation from the 500 nm thick LN sample. The recorded time domain signals are shown in Figure 2b for different combinations of pump and THz polarizations. The time traces show the primary pulses generated in the thin LN film and echo pulses due to reflections in the GaP detection crystal. By evaluating the Fourier transform of the time domain signals, we retrieve the power density spectra of the generated THz radiation that are shown in Figure 3a–d. The polarization-resolved measurements reveal different line shapes and allow us to disentangle the contributions of the different tensorial components of . We observe that the generated THz radiation polarized along the *z*-axis (*zzz* and *zyy*) features a wider spectrum, whereas THz signals polarized along the *y*-axis (*yyy* and *yzy*) show a more pronounced peak at 4 THz and a dip at 6 THz. These are clear signatures of the ionic enhancement of the nonlinear response in the LN film and are due to the combination of the nonlinear response dispersion (Figure 1b), the LN absorption in the THz band (Figure 1a), the spectral response of the GaP electro-optic sampling (see Supporting Information S2), and the spectral band of the pump pulse which has a peak for optical rectification at 5.2 THz (see Supporting Information S4).

To corroborate the key role of the ionic contribution to the nonlinear response, we developed numerical calculations in the frequency domain based on the finite-element method implemented in COMSOL to analyze the optical-to-THz conversion process. The complex permittivity of LN is modeled according to eq 1 in the THz band and with a Sellmeier equation for the optical band.^16^ The nonlinear polarization generated at the frequency Ω by optical rectification of a pulse centered at frequency ω_0_ is given by
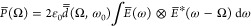
5where  is the electric field of the optical pulse. The spectra of the generated THz radiation that result from numerical calculations are shown in Figure 3a–d overlapped to experimental data and normalized to the signal obtained in the *zzz* polarization configuration. We find an excellent agreement between experimental and numerical results by re-scaling the magnitude of the nonlinear coefficients obtained from theory relative to the *d*_33_ element. These scaling parameters are equal to 2 for *d*_22_, 0.9 for *d*_31_, and 0.32 for *d*_15_. This difference might be due to experimental uncertainties of our experimental setup and of the values of the low-frequency nonlinear coefficients^20^ used to fit eq 3, as well to defects and impurities in the LN crystal structure. The excellent agreement between experimental and numerical results clearly indicates the key role of the ionic contribution to the nonlinear response. Indeed, had we neglected the dispersion of the nonlinear coefficients, we would have observed a reduction of the THz signal generated at frequencies close to the phonon resonances (see Supporting Information S5).

**Figure 4:**
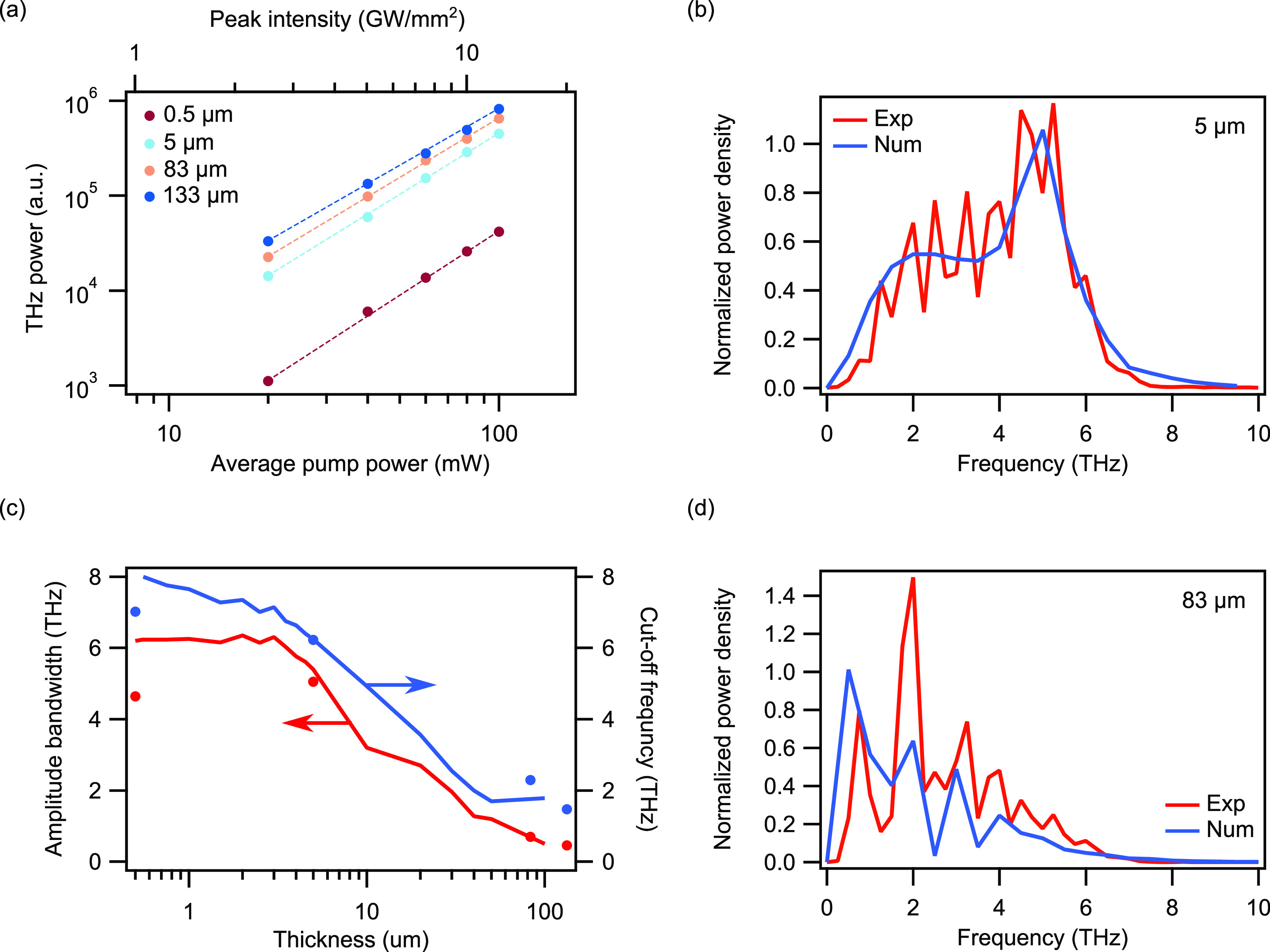
(a) Measured power of generated THz radiation as a function of pump power and film thickness in the *zzz* pump-emission polarization configuration. The experimental data are fitted with straight dashed lines, each characterized by a slope of 2.15, 2.11, 2.06, and 2.09, for film thicknesses of 0.5, 5, 83, and 133 μm, respectively. (c) FWHM bandwidth (left-axis) and high-frequency cutoff (right-axis) of the amplitude spectrum of the generated THz radiation. Continuous lines show numerical results while filled dots show experimental data. (b,d) THz emission spectra from 5 and 83 μm bulk samples, respectively. The spectra derived from the measurements are normalized to the maximum value after a moving average filter is applied.

To further confirm that the THz signal observed in the experiments is generated by optical rectification in the thin LN film, we performed optical rectification measurements at different average pump powers and on LN samples with different thicknesses. We chose the *zzz* combination of the optical pulse and THz radiation polarizations. The power of the generated THz signal is derived by integration of the THz spectral density over all frequencies and it is shown in Figure 4a. As expected for second-order nonlinear processes, the power of the generated signal increases quadratically with the pump power. On the other hand, at a constant pump power, the signal increase by varying the LN sample thickness shows a strong deviation from a quadratic law due to absorption of the THz radiation in LN (see Supporting Information S6). Figure 4b,d report the THz spectra obtained from the measurements and the numerical simulations on a 5 μm LN film and an 83 μm bulk LN sample. Analyzing these results with the ones in Figure 3a, we observe that when the LN thickness is increased, the spectral power density in the low-frequency band becomes dominant and obscures the THz generation above 4 THz. Furthermore, numerical results reported in Figure 4c show that the full-width at half-maximum (FWHM) bandwidth of the amplitude spectra remains constant at a value of about 6.2 THz for LN film thickness between 0.5 and 3.5 μm and it rapidly decreases when the LN film thickness is increased above 3.5 μm. The measurements, represented with dots in Figure 4c, are in good agreement with the numerical prediction. The dependence of the bandwidth of the generated THz signal on the LN film thickness is due to the increased absorption that limits the effective length (see Figure 1c). Indeed, the high-frequency cutoff shown in Figure 4c, right *y*-axis, rapidly decreases from a maximum frequency of 8 to 0.2 THz when the LN film thickness is varied from 0.5 to 100 μm. This quenches the optical-to-THz conversion efficiency in the absorption band of LN, resulting in a significant reduction of the emission bandwidth and cutoff frequency. Thus, from these observations, we can conclude that the optical-to-THz conversion in thin LN films is enhanced by PhP despite operating in a strong absorption band for the LN.

As shown in Figure 1a, the real part of the permittivity of LN can assume negative values in the THz band as an effect of the PhP. In this range, quasi-bound electromagnetic modes at the surface may be supported and, when LN is nanostructured, localized surface phonon–polariton (LSPhP) modes can manifest.^25,28^ These modes are similar to plasmonic excitations at optical frequencies and, interestingly, they can be engineered to tune and enhance the THz generation process.^7,29^ To illustrate this potential, we use numerical calculations in COMSOL to examine the optical-to-THz conversion in a sub-wavelength LN rod suspended in air.

**Figure 5:**
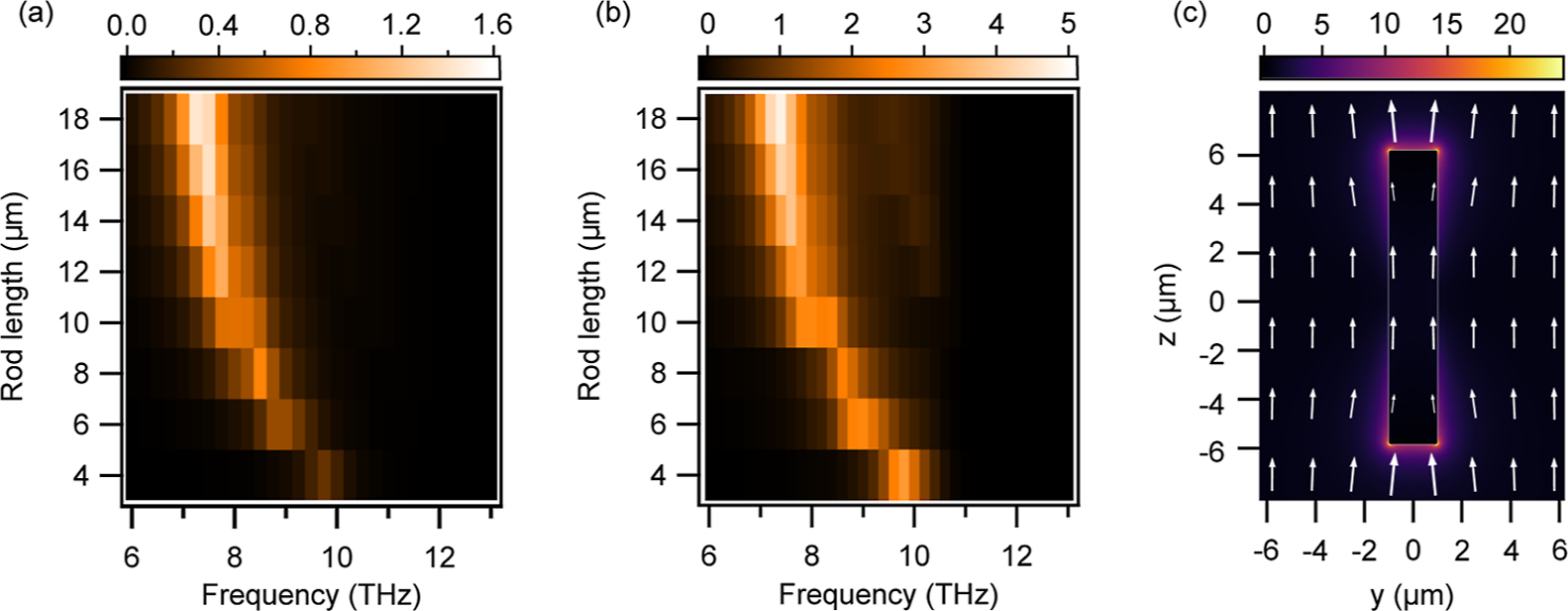
(a) Scattering efficiency of LN bar as a function of bar length evaluated by numerical simulations. (b) THz generation efficiency normalized to unstructured thin film evaluated by numerical simulations. (c) Electric field enhancement in the *yz*-plane crossing the LN rod at the LSPhP resonance (rod length = 12 μm, rod width = 2 μm, rod height = 500 nm, Ω = 7.75 THz). The white arrows show the in-plane electric field vector.

The scattering efficiency spectrum of an LN rod in a homogeneous environment of air is shown in Figure 5a. The rod axis is parallel to the *e*-axis and it is illuminated by a THz electric field polarized along the same direction. We exploit the problem’s symmetry to reduce the computational domain, focusing on a quarter section of the rod. This portion is defined by two orthogonal mirror planes: one parallel and one orthogonal to the rod’s long axis. On the plane parallel to the rod’s long axis, we implement a perfect magnetic conductor boundary condition, while on the plane orthogonal to the rod’s long axis we enforce a perfect electric conductor boundary condition. We fix the rod thickness and width to 0.5 and 2 μm, respectively, and study the response in the THz band as a function of the rod length. The scattering spectrum is limited to the region of negative permittivity of LN and it exhibits a peak that moves towards shorter frequencies when the rod length is increased. The high-frequency limit is well captured by the Fröhlich condition ε_r_(Ω_0_) = −2. As the rod length increases, retardation effects cause an energy shift of the resonance that resembles the behavior of plasmonic sub-wavelength antennas.^30^

Figure 5b reports the THz spectrum generated by optical rectification of a 50 fs pump beam at a wavelength of 1500 nm as a function of the LN rod length. The power of the THz radiation emitted in the forward direction with respect to the pump beam propagation is divided by the geometrical cross-section of the rods (i.e., σ = *l* × *w*). This THz intensity is normalized to the one obtained by an LN film of the same thickness. We observe that the peak of the THz spectrum closely follows the peak of the scattering efficiency of the LN rod. Furthermore, as the linear scattering efficiency in the THz band is increased by the rod, the optical-to-THz conversion is enhanced by almost a factor of 5 compared to the unstructured film. These results show the strong potential of phonon-resonances engineering to tailor the properties of the generated THz radiation at a sub-wavelength scale.

## Conclusions

We experimentally demonstrate, for the first time to our knowledge, strong enhancement of optical nonlinearity from 2 to 8 THz in an LN thin film due to phonon–polaritons. Polarization-resolved measurements provide essential insight into the tensorial nature of the nonlinear susceptibility. Based on theoretical models that take into account the ionic contribution, we perform numerical calculations of the THz generation process that are in excellent agreement with the measurements and allow us to estimate the relative magnitude and PhP enhancement of all the second-order nonlinear coefficients of LN. Furthermore, we theoretically propose structuring the LN thin film on the THz sub-wavelength scale to engineer LSPhP resonances that could further enhance and control the frequency, bandwidth, direction, and polarization of the THz emission.^25,29^ Our demonstration unlocks the possibility for the development of THz-optical metasurfaces for the generation and detection of highly broadband-tailored THz radiation based on the TFLN material system.

## Supporting Information Available

List of the parameters used to model the complex relative permittivity of LN in the THz band. Nonlinear polarization combinations. Calculation of optical rectification bandwidth. Role of nonlinear coefficient dispersion in the optical rectification process. THz power vs LN thickness (PDF)
