# Supplementary material for: Nonlinear THz Generation through Optical Rectification Enhanced by Phonon–Polaritons in Lithium Niobate Thin Films
Source: ACS Photonics. 2023 Aug 28;10(9):3419–25. doi: 10.1021/acsphotonics.3c00924 (PMC10515699; doi:10.1021/acsphotonics.3c00924)
Supplement: Supplementary file 1 — ph3c00924_si_001.pdf [file ph3c00924_si_001.pdf]

Nonlinear THz generation through optical rectification enhanced by phonon-polaritons in lithium niobate thin films

Luca Carletti,[*,†,‡,⊥] Cormac McDonnell,[¶,⊥] Unai Arregui-Leon,[§] Davide Rocco,[†,‡] Marco Finazzi,[§] Andrea Toma,[‖] Tal Ellenbogen,[¶] Giuseppe Della Valle,[§] Michele Celebrano,[§] and Costantino De Angelis[†,‡]

†*Department of Information Engineering, Univeristy of Brescia, Via Branze 38, 25123, Brescia, Italy*

‡*National Institute of Optics - National Research Council (INO-CNR), Via Branze 45, 25123, Brescia, Italy*

¶*Department of Physical Electronics, Fleischman Faculty of Engineering, Tel-Aviv University, 69978, Tel-Aviv, Israel*

§*Department of Physic, Politecnico di Milano, Piazza Leonardo da Vinci 32, 20133, Milano, Italy*

‖*Istituto Italiano di Tecnologia, 16163, Genova, Italy*

⊥*These authors contributed equally to this work.*

E-mail: luca.carletti@unibs.it

S1 Parameter values for complex relative permittivity

The parameters used to evaluate the complex relative permittivity with the model of Eq. 1 of the main text are obtained from ref.[1] and are here reported in Table S1 and S2 for the

e-axis and the o-axis, respectively. The high-frequency permittivity limit, ε_∞, is equal to 4.6 and 5 for e-axis and o-axis, respectively.

Table S1: Parameters of e-axis PhP.

ω_T (cm^{-1})	S	Γ (cm^{-1})
248	16	21
274	1	14
307	0.16	25
628	2.55	34
692	0.13	49

Table S2: Parameters of o-axis PhP.

ω_T (cm^{-1})	S	Γ (cm^{-1})
152	22	14
236	0.8	12
265	5.5	12
322	2.2	11
363	2.3	33
431	0.18	12
586	3.3	35
670	0.2	47

S2 Description of the experimental setup

Pump probe time domain spectroscopy was used detect the temporal and spectral properties of the generated THz pulses emitted from the LN thin films. The laser source used was an ultrashort femtosecond laser (Spectra Physics Solstice Ace) emitting pulses at a central wavelength of 800 nm with a pulse duration of approximately 35 fs. The output beam of the laser was split into pump and probe arms using a 99:1 beam splitter. On the pump arm, the beam was directed into an optical parametric amplifier were an output wavelength of 1500 nm was chosen for the experiments. A number of optical elements were used to control the laser power and final polarization angle. A convex lens (f = 1000 mm) was used to focus the pump beam onto the sample, with a spot diameter of ≈600 µm. The LN sample was

placed at the focal point of an off axis parabolic mirror (f = 50.8 mm) which was used to collect and collimate the generated THz beam. A second off-axis parabolic mirror was used to focus the collected THz beam into a GaP nonlinear crystal (t =100 µm) for electro-optic sampling (see Fig. S1 for the response function). On the probe line the ultrashort pulse was sent to a motorized delay stage which was used to control the temporal position of the probe pulse. Then the probe was directed through a small hole in the second parabolic mirror where it was spatially and temporally overlapped with the generated THz pulse. The temporal electric field amplitude and phase was measured using electro-optic detection, consisting of a quarter wave plate, a Wollaston prism and a balanced photodiode. The photodiode signal was amplified using a lock-in detector with an integration time of τ = 300 ms, which was synchronized to the laser source using a mechanical chopper at 1 kHz. In order to optimize the detected bandwidth and minimize THz absorption in the humid air, the sample, collection and detection components were placed in an enclosure and pumped with dry air down to a humidity of less than 1%. Typical variations of the measured THz electric field amplitude using this setup are of the order of 3-4%.

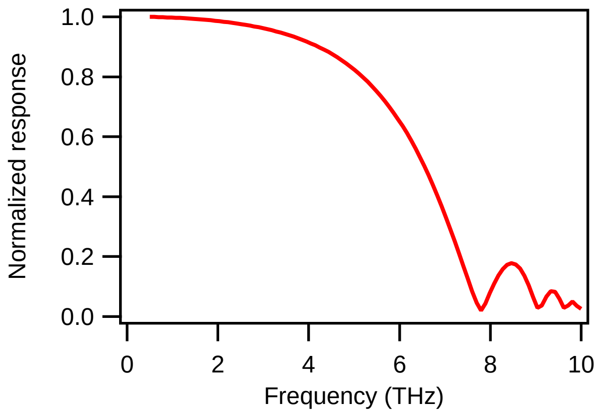

Figure S1: Normalized transfer function of the electro-optic detector based on 100 µm thick GaP crystal.

S3 Nonlinear polarization combinations

The nonlinear polarization generated at the THz frequency Ω in the configurations listed in the text are here described:

Pump polarized along the e-axis (z-axis) of LN:

$$P_z(\Omega) \propto d_{33} E_z E_z^* \tag{1}$$

$$P_y(\Omega) = 0 \tag{2}$$

Pump polarized along the o-axis (y-axis) of LN:

$$P_z(\Omega) \propto d_{31} E_y E_y^* \tag{3}$$

$$P_y(\Omega) \propto d_{22} E_y E_y^* \tag{4}$$

Pump polarized at 45° from the e-axis of LN:

$$P_z(\Omega) \propto d_{31} E_y E_y^* + d_{33} E_z E_z^* \tag{5}$$

$$P_y(\Omega) \propto d_{22} E_y E_y^* + d_{15} E_y E_z^* \tag{6}$$

S4 Optical rectification bandwidth

The bandwidth of the signal generated by optical rectification of a laser pulse, ignoring the material response, can be estimated from its temporal profile. Assuming $S(t)$ the temporal envelope of the pulse, its Fourier transform, $\tilde{S}(\omega)$, provides the spectral density. The nonlinear currents at a frequency Ω due to optical rectification of the laser pulse are thus

$$\tilde{J}(\Omega) = -i\Omega \int \tilde{S}(\omega)\tilde{S}(\omega - \Omega) d\omega \tag{7}$$

In the experiments, a Gaussian shaped optical pulse with a duration of 50 fs (full-width at half-maximum) and centered at a wavelength of 1500 nm is used. By applying Eq. 7 we obtain a bandwidth of $\tilde{J}(\Omega)$ of about 9 THz with a peak at 5.2 THz.

S5 Role of nonlinear coefficient dispersion in the optical rectification process

The spectrum of the THz signal generated by optical rectification in the LN films and crystals shows characteristic features that are to attribute to the phonon-polariton enhancement of the material nonlinearity. To emphasize once more the role of the phonon-polariton and the ionic contribution to the nonlinear response we compared the signal generated in the zzz and yyy configuration in a 500 nm thick LN film calculated in numerical simulations in which the nonlinear coefficient dispersion is either considered or neglected. As it can be seen in Fig. S2, for constant nonlinear coefficients, the spectrum of the generated THz signal for the zzz polarization configuration (see Fig. S2(a)) shows a narrower bandwidth than when the ionic contribution to the nonlinear coefficient is considered. This is due to the sole effect of increased absorption with frequency in LN. Furthermore, we observe a larger difference in the yyy polarization configuration in Fig. S2(b). When the $d22$ dispersion is neglected, the THz spectrum depends only on the absorption spectrum that indeed shows a peak at the frequency of 4.5 THz. On the other hand, when the ionic contribution to the nonlinear response is considered, we observe a peak of the generated THz signal in this band which is in excellent agreement with the experimental results.

S6 THz power vs LN thickness

The dependence of the power of the emitted THz electromagnetic radiation as a function of the LN thickness is shown in Fig. S3(a). The power experiences a rise with an increase in

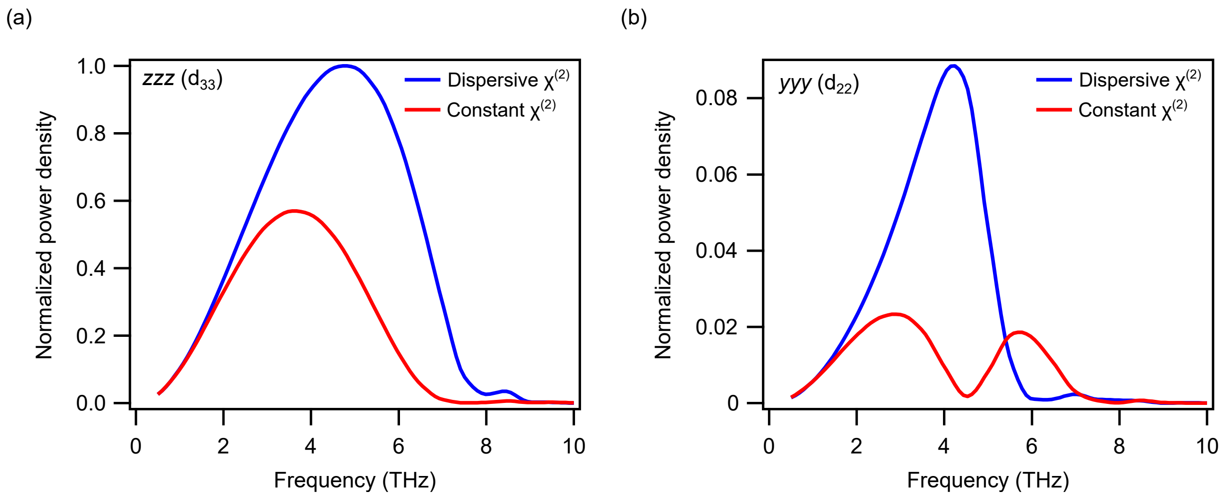

Figure S2: (a-b) THz emission spectra from a LN film of 500 nm thickness calculated numerically either accounting for the ionic contribution to the nonlinear response (Dispersive $\chi^{(2)}$) or assuming a constant value of the nonlinear coefficient over the full THz band (Constant $\chi^{(2)}$). The last two letters indicate the optical laser pulse polarization with respect to the LN axes, the first letter indicates the polarization of the emitted THz radiation.

LN thickness because the THz signal at low frequencies augments. Due to the absorption induced by the coupling with the phonon-polariton, the signal generated close to the phonon resonance shows a less pronounced increase as a function of the crystal thickness than the low-frequency band where absorption is negligible, as observed in Fig.S3(b). In particular, for a thickness of 5 µm, the optical rectification peak of the pump beam becomes dominant due to the combined effect of THz absorption and ionic enhancement of the nonlinear response. In Fig. S4, we show the power of the signal generated in correspondence to the e-axis phonon resonance (7.45 THz). We observe that the power first increases when the LN thickness is increased and then it saturates when the LN thickness is above 2 µm. This is in agreement with the effective length calculation reported in Fig. 1(c) of the main text and is due to the strong absorption caused by the PhP.

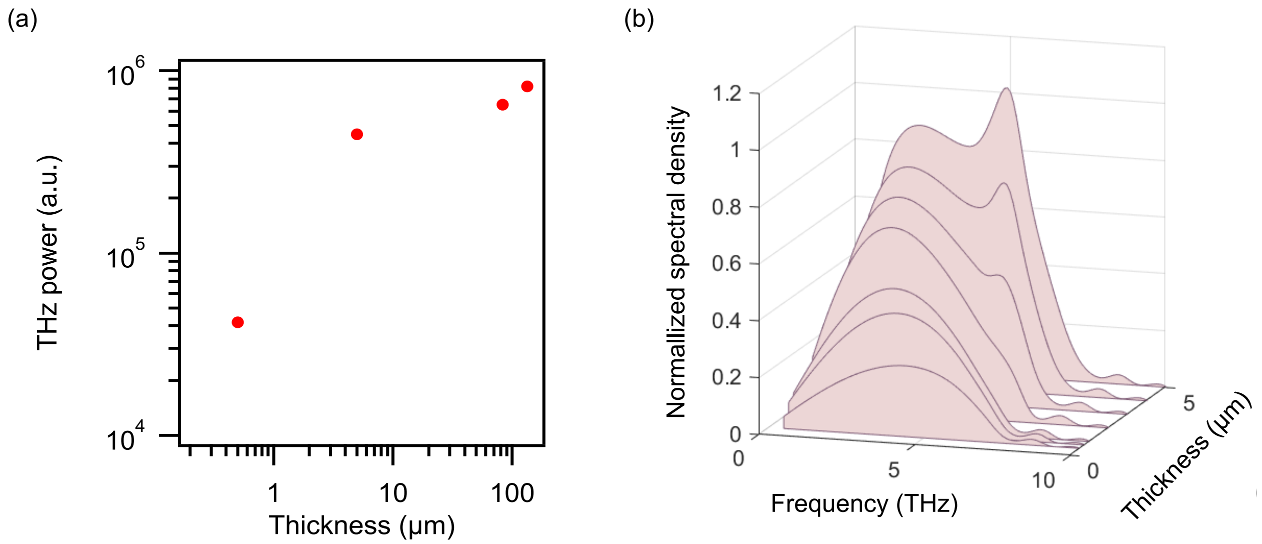

Figure S3: (a) Power of the generated THz radiation as a function of LN film thickness. The pump average power is constant at 100 mW. (b) Evolution of the THz signal spectrum as a function of the LN thickness.

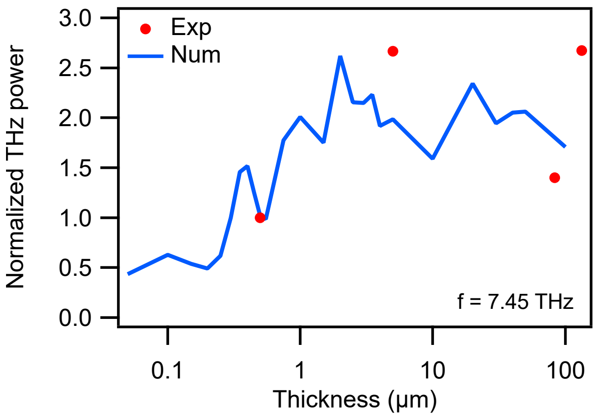

Figure S4: Power of the generated THz radiation at the e-axis phonon frequency of 7.45 THz as a function of LN film thickness. The pump average power is constant at 100 mW.

References

(1) Barker, A. S.; Loudon, R. Dielectric Properties and Optical Phonons in $LiNbO_3$. *Physical Review* **1967**, *158*, 433–445.
